# Gastrointestinal Microbiome Dysbiosis in Infant Mice Alters Peripheral CD8^+^ T Cell Receptor Signaling

**DOI:** 10.3389/fimmu.2017.00265

**Published:** 2017-03-08

**Authors:** Gabriela Gonzalez-Perez, Esi S. N. Lamousé-Smith

**Affiliations:** ^1^Division of Pediatric Gastroenterology, Hepatology and Nutrition, Department of Pediatrics, Columbia University Medical Center, New York, NY, USA

**Keywords:** microbiome, dysbiosis, antibiotics, CD8^+^ T cells, T cell signaling, Erk2, neonatal, immunity

## Abstract

We recently reported that maternal antibiotic treatment (MAT) of mice in the last days of pregnancy and during lactation dramatically alters the density and composition of the gastrointestinal microbiota of their infants. MAT infants also exhibited enhanced susceptibility to a systemic viral infection and altered adaptive immune cell activation phenotype and function. CD8^+^ effector T cells from MAT infants consistently demonstrate an inability to sustain interferon gamma (IFN-γ) production *in vivo* following vaccinia virus infection and *in vitro* upon T cell receptor (TCR) stimulation. We hypothesize that T cells developing in infant mice with gastrointestinal microbiota dysbiosis and insufficient toll-like receptor (TLR) exposure alters immune responsiveness associated with intrinsic T cell defects in the TCR signaling pathway and compromised T cell effector function. To evaluate this, splenic T cells from day of life 15 MAT infant mice were stimulated *in vitro* with anti-CD3 and anti-CD28 antibodies prior to examining the expression of ZAP-70, phosphorylated ZAP-70, phospho-Erk-1/2, c-Rel, total protein tyrosine phosphorylation, and IFN-γ production. We determine that MAT infant CD8^+^ T cells fail to sustain total protein tyrosine phosphorylation and Erk1/2 activation. Lipopolysaccharide treatment *in vitro* and *in vivo*, partially restored IFN-γ production in MAT effector CD8^+^ T cells and reduced mortality typically observed in MAT mice following systemic viral infection. Our results demonstrate a surprising dependence on the gastrointestinal microbiome and TLR ligand stimulation toward shaping optimal CD8^+^ T cell function during infancy.

## Introduction

Experimental studies continue to offer robust evidence that the gastrointestinal microbiome (GIM) exerts a major influence on anatomic and cellular development of the immune system and function (1–3). Colonization of the intestine by specific bacteria stabilizes immune regulatory cell populations implicated in maintaining health. For example, Th1/Th2 balance (4), Th17 (5), Treg (6), and mucosal iNKT cells (7) have been shown to require the GIM for their normal development and function. Thus, disrupting the natural progress of intestinal colonization that occurs in the infant GIM may have both immediate and long-lasting immune consequences. Aggressive standards of hygiene, antibiotic use, and malnutrition cause significant short- and long-term disruption on the assembly and maturation of the infant GIM (8, 9). We are now appreciating that the immune effects of GIM dysbiosis may not be realized in the short term. There are emerging high quality studies that implicate early-life antibiotic use with the enhanced risk of developing allergic and autoimmune diseases later in life (10–14). However, the corresponding impact of GIM dysbiosis on specific parameters of infant T cell function has not been extensively assessed.

The GIM of infants is particularly vulnerable due to its inherent instability and lower species diversity in comparison with older children and adults (15, 16). Antibiotics are the most commonly prescribed medication in pregnant woman and children and thus a leading cause of intestinal dysbiosis. Up to 40% of pregnant women are treated with antibiotics during the perinatal period (17, 18) and up to 75% of children have been treated with at least one course of antibiotics before the age of 2 (19, 20). The most common antibiotic treatment of pregnant women is during labor with intrapartum antibiotic prophylaxis (IAP) to prevent perinatal group B streptococcal (GBS) disease in infants. Penicillin, ampicillin, vancomycin, or clindamycin are common antibiotics used to treat GBS-colonized mothers during labor (21). Several studies have detailed the long-term impact of even short courses of antibiotics on the density, complexity, and stability of the GIM (22, 23). The effect of maternal intrapartum antibiotics on the infant GIM has been detailed in a handful of studies and using a culture and non-culture based techniques (24–27). To our knowledge, no studies have evaluated how IAP induced dysbiosis of the infant GIM can alter the function of individual adaptive cellular immune subsets following birth.

Infants are characterized by a distinct GIM. Therefore, the functional behavior and phenotype of infant immune effectors may not be regulated by the GIM in the same way as has been demonstrated in adults. Infant T cells are compromised in their capacity to regulate interferon gamma (IFN-γ), an important mediator in controlling viral and other intracellular pathogen infections. As demonstrated in mice and humans, infant T cells are capable of producing IFN-γ in response to infection, but this may be skewed in timing (too early) and in magnitude (too weak) as compared to adult T cells (28). These developmental differences may be modulated by intrinsic or extrinsic mechanisms and include epigenetic hypermethylation of the IFN-γ promoter (29), increased T cell receptor (TCR) activation threshold regulated by miRNA (30), inhibition by Tregs (31), skew toward innate functional profile (32), insufficient facilitation by infant antigen-presenting cells (33), or by metabolites of the GIM (34, 35).

We have demonstrated that infant mice with GIM dysbiosis caused by maternal antibiotic treatment (MAT) in the last days of pregnancy confer increased susceptibility to death following a systemic viral infection (36). We observe that CD8^+^ effector T cells from MAT infant mice consistently demonstrate the inability to sustain IFN-γ production *in vivo* following vaccinia virus infection and *in vitro* upon TCR stimulation. The goals of this study were to further evaluate the intrinsic functional capacity of MAT CD8^+^ T cells and assess proximal and distal signaling molecule activation following TCR engagement. We further determined whether transfer of MAT CD8^+^ T cells into a non-dysbiotic host environment or stimulation with lipopolysaccharide (LPS) was sufficient to rescue signal activation and IFN-γ responsiveness of MAT CD8^+^ T cells.

## Materials and Methods

### Mice

All animal studies were conducted in compliance with an animal protocol approved by the Institutional Animal Care and Use Committee of Columbia University Medical Center. Six- to eight-week-old C57BL/6J, OT-I CD45.1, Rag1KO (B6.129S7-*Rag1^tm1Mom^*/J), MyD88KO [B6.129P2(SLJ)-*Myd88^tm1.1Def^*/J], and TLR4KO (B6.B10ScN-*Tlr4^lps-del^*/JthJ) mice were housed for at least 7 days prior to breeding and were maintained under specific pathogen-free BSL-1 and BSL-2 conditions in Columbia University Medical Center animal facilities. Breeding pairs were set up in harem, two females and one male per cage. Pregnant females were separated from males and single housed at day 17 postmating. Mice transferred between facilities were allowed to acclimate for ≥1 week prior to initiation of experiments. OT-I CD45.1 mice were derived by crossing OT-I [C57BL/6-Tg (TcrαTcrβ)1100Mjb/J] and CD45.1 (B6.SJL-*Ptprc^a^Pepc^b^*/BoyJ) mice for more than 10 generations. Phenotype of OT-I CD45.1 mice was confirmed by flow cytometry with antibodies specific for CD45.1 (clone A20), CD8α (clone 53-6.7), TCR Vα2 (clone B20.1) and TCR V5.1 (clone MR9-4), all from BioLegend (San Diego, CA, USA). All parental strains were obtained from the Jackson Laboratory (Bar Harbor, ME, USA). Female and male pre-weaning littermates were used in all experiments.

### Maternal Antibiotic Treatment

Pregnant mothers were allowed to drink antibiotic-treated water containing a cocktail of ampicillin (AuroMedics), streptomycin (X-Gen Pharmaceuticals), and clindamycin (Aurobindo Pharma) mixed in sterile water (each at 1 mg/ml) *ad libitum* 3–5 days prior to birth of a litter and for the duration of the experiments. The antibiotic-treated water was replaced every 3 days. We previously determined that it takes only 3 days to alter the microbiome significantly using this cocktail (data not shown) and that refreshing the antibiotic solution every 3 days maintains durable depletion of the microbiota in adult mice.

### T Cell Isolation and *In Vitro* Activation Assays

For the *in vitro* LPS stimulation and OT-I transfer experiments, total CD8^+^ T cells were purified from gender-matched pooled spleens of 15-day-old control (CTRL) and MAT littermate C57BL/6J, MyD88KO, TLR4KO, and OT-I mice, respectively, using the MojoSort mouse CD8 isolation kit (BioLegend) plus biotin anti-CD71 (clone RI7217), biotin anti-CD45R/B220 (clone RA3-6B2), and biotin anti-TER119 (clone TER119) followed by MACS negative selection (Miltenyi Biotec). CD8^+^ T cell purity routinely yielded 98%. To assess the effect of LPS treatment on CD8^+^ T cell cytokine production, CD8^+^ T cells were stimulated with plate bound anti-CD3 (1 μg/ml; clone 145-2C11) and soluble anti-CD28 (2 μg/ml; clone 37.51) with or without *Escherichia coli* 055:B5-derived LPS (1 μg/ml; InvivoGen) for 72 h. For the TCR signaling assays, total T cells were enriched from individually processed spleens of 15-day-old CTRL and MAT littermate C57BL/6J mice using biotin anti-CD71 (clone RI7217), biotin anti-CD45R/B220 (clone RA3-6B2), and biotin anti-TER119 (clone TER119) followed by MACS negative selection (Miltenyi Biotec). To generate effector T cells for TCR signaling assays, total T cells (2 × 10^5^ cells/200 μl) were stimulated with plate bound anti-CD3 (1 μg/ml; clone 145-2C11) and soluble anti-CD28 (2 μg/ml; clone 37.51) in RPMI-10 (RPMI 1640 supplemented with 10% FBS, 20 mM HEPES, 2 mM l-glutamine, 0.1 mM 2-mercaptoethanol, 50 μg/ml gentamicin sulfate, 50 U/ml penicillin, and 50 μg/ml streptomycin) in 96 well flat bottom plates and incubated at 37°C with 5% CO_2_ for 24, 48, and 72 h. In some TCR signaling assays, purified total CD8^+^ T cells were used as indicated. All antibodies were from BioLegend.

### OT-I Adoptive Cell Transfer Experiments

Control and MAT OT-I CD8^+^ T cells pooled from littermates (1.5 × 10^5^/100 μl PBS) were transferred into age- and gender-matched CTRL Rag1KO recipients by intraperitoneal (i.p.) injection. Twenty-four hours after OT-I adoptive cell transfer, recipient mice were infected i.p. with 1 × 10^4^ PFU of recombinant vaccinia-ovalbumin (Vac-OVA) by i.p. injection. Mice were monitored daily for weight loss and appearance of illness. Eight days after infection, mice were euthanized by CO_2_ inhalation. Peritoneal exudate cells (PEC) were aspirated following lavage of the peritoneum with 1 ml sterile PBS. Spleens and mesenteric lymph nodes (MLN) were mechanically disrupted to obtain single cell suspensions and then treated with ACK buffer to lyse red blood cells. For the detection of cytokines, the cells were cultured for 5 h in RPMI-10 with SIINFEKL peptide (5 μM; New England Peptide) in the presence of brefeldin A and monensin (BioLegend).

### *In Vivo* LPS Treatment and Vac-OVA Infection

Fifteen-day-old CTRL and MAT C57BL/6J infant mice were infected with Vac-OVA (1 × 10^4^ PFU i.p.) and orally treated with *E. coli* 0111:B4-derived LPS (50 μg orogastric; InvivoGen) beginning on the day of the infection and continuing every other day for 10 days. Mice were monitored daily for weight loss and appearance of illness. Eleven days after infection, mice were euthanized by CO_2_ inhalation. Spleens were mechanically disrupted to obtain single cell suspensions and then treated with ACK buffer to lyse red blood cells. For the detection of cytokines, the splenocytes were cultured for 5 h in RPMI-10 with SIINFEKL peptide (5 μM; New England Peptide), phorbol 12-myristate 13-acetate (PMA) (10 ng/ml), and ionomycin (1 μg/ml) in the presence of brefeldin A and monensin (BioLegend).

### Flow Cytometry

Single cell suspensions of lymphocytes isolated from the spleen, MLN, or PEC of uninfected and infected infant mice were stained with optimal concentrations of the following antibodies and reagents: CD3ε (clone 145-2C11), CD8α (clone 53-6.7), CD25 (clone PC61), CD44 (clone IM7), CD62L (clone MEL-14), CD69 (Clone H1.2F3), TNF-α (clone MP6-XT22), IFN-γ (clone XMG1.2), pTyr (clone pY20), ZAP-70 (clone 1E7.2), phosphorylated ZAP-70 (pZAP-70) Tyr319 (clone n3kobu5; eBioscience), Erk2 (clone REA186; Miltenyi Biotec), phospho-Erk-1/2 (pErk1/2) Thr202/Tyr 204 (clone 4B11B69), c-Rel (clone REA397; Miltenyi Biotec), Ki-67 (clone B56; BD Biosciences), annexin V, and 7-aminoactinomycin D (7-AAD). All antibodies and reagents were from BioLegend unless otherwise specified. Dead cells were excluded from the analysis by staining with Zombie Aqua (BioLegend). Cells were analyzed on a Fortessa flow cytometer (Becton Dickinson) using CellQuest™ software. Data were analyzed using FlowJo v10 analysis software (TreeStar).

### TCR Signaling Analysis by Flow Cytometry

Infant CTRL and MAT T cells (5 × 10^5^ cells/50 μl) that were freshly isolated (unstimulated) or stimulated with anti-CD3/anti-CD28 for 24, 48, and 72 h were incubated with or without soluble anti-CD3 (10 μg/ml; clone 145-2C11; BioLegend) and soluble anti-CD28 (10 μg/ml; clone 37.51, BioLegend) in cold RPMI 1640 supplemented with 0.5% FBS in 96 well round bottom plates at 4°C for 15 min, washed with cold medium, incubated with or without soluble goat anti-hamster IgG (20 μg/ml; Jackson ImmunoResearch Laboratories) at 4°C for 15 min, washed with cold medium, then resuspended in 50 μl of medium and incubated in a 37°C water bath for 2 min. After CD3/CD28 crosslinking, cells were immediately fixed with 50 μl of pre-warmed Cytofix buffer (BD Biosciences) for Erk2 and pErk1/2 staining or IC Fixation buffer (eBioscience) for ZAP-70, pZAP-70, pTyr, and c-Rel staining, incubated at 37°C for 10 min, and washed with FACS buffer (HBSS containing 1% FBS and 0.1% sodium azide) prior to staining with antibodies for CD8α, CD44, and CD62L at 4°C for 30 min. For intracellular staining of Erk2 and pErk1/2, cells were permeabilized with pre-chilled Phosflow Perm III buffer (BD Biosciences). For intracellular staining of ZAP-70, pZAP-70, pTyr and c-Rel, cells were permeabilized with Perm Wash buffer (BioLegend). Cells were incubated with intracellular antibodies at 4°C overnight, washed with Perm Wash buffer and then FACS buffer prior to analysis. Analysis was performed gating on naïve (CD44^−^) or effector (CD44^+^) CD8^+^ T cells as indicated.

### Statistical Analysis

Statistical analysis was performed using GraphPad Prism 7.0. Data were analyzed by one-way ANOVA with Holm–Sidak posttest or unpaired two-tailed Student’s *t*-test as indicated. In all analyses, values with *p* < 0.05 were considered statistically significant.

## Results

### MAT Effector CD8^+^ T Cells Exhibit Less Polyfunctional Cytokine Responses than CTRL Cells

We recently reported that infant mice whose mothers were treated with antibiotics (MAT) in the last days of pregnancy and during lactation have significantly altered composition of the GIM, enhanced susceptibility to systemic viral infection, altered innate immune cell populations, and poor effector CD8^+^ T cell responses compared with CTRL infants (36). Particularly, MAT effector CD8^+^ T cells are unable to sustain IFN-γ production *in vivo* following vaccinia virus infection and *in vitro* upon TCR and CD28 stimulation (36). These findings led us to hypothesize that the dysfunction observed in MAT CD8^+^ T cells was due to intrinsic cell defects.

**Figure 1:**
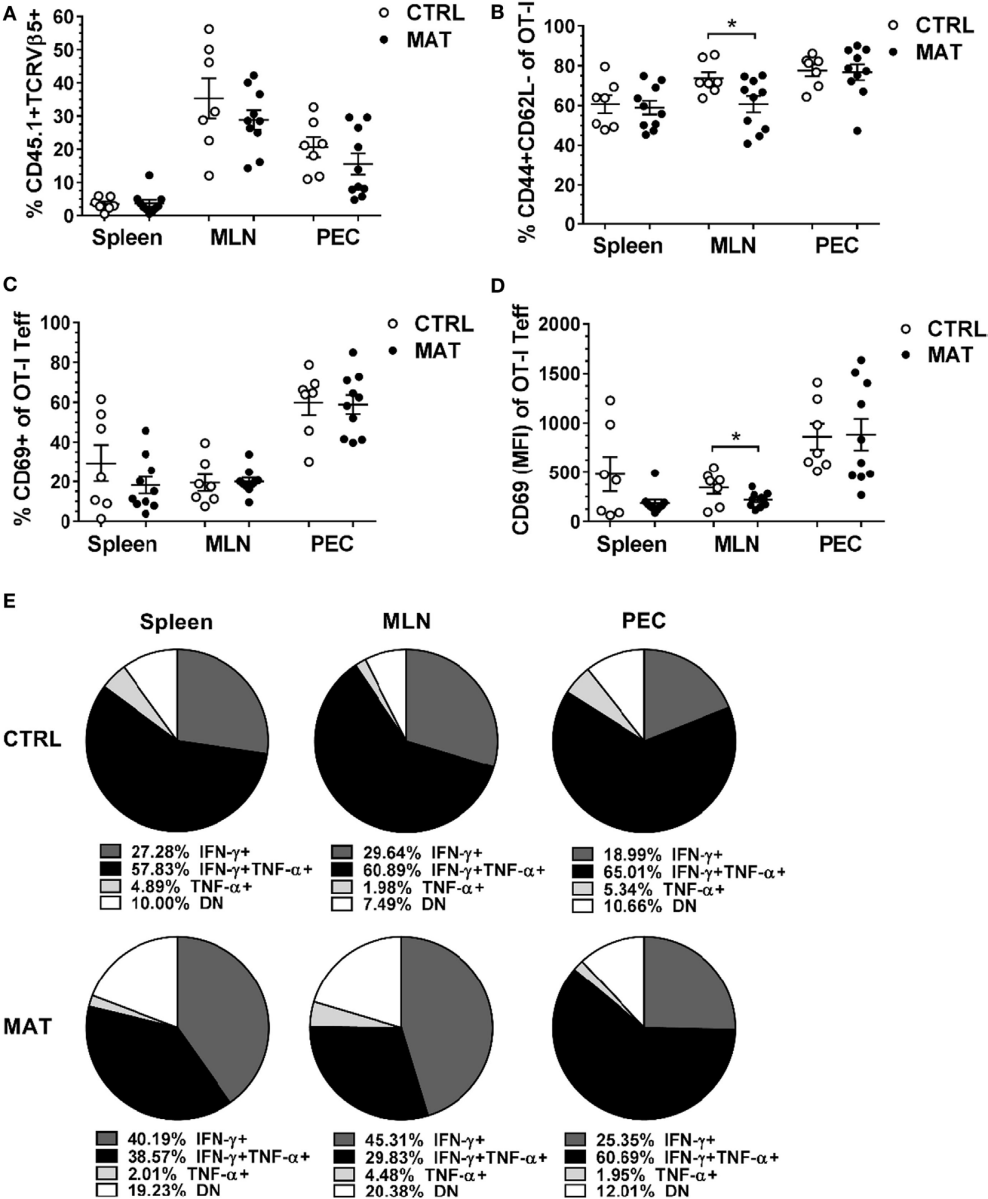
**Maternal antibiotic treatment (MAT) effector CD8^+^ T cells exhibit less polyfunctional cytokine responses than control (CTRL) effector cells**. Congenic (CD454.1) OT-I CD8^+^ T cells isolated from the spleens of day of life 15 CTRL and MAT infant mice were adoptively transferred into age- and gender-matched CTRL Rag1KO recipient mice 24 h prior vaccinia-OVA infection (1 × 10^4^ PFU intraperitoneal). Lymphocytes isolated from the spleen, mesenteric lymph nodes (MLN), and peritoneal exudate cells (PEC) of CTRL Rag1KO recipient mice were analyzed for **(A)** percentage of CD45.1 and T cell receptor (TCR) Vβ5^+^ cells of total lymphocytes, **(B)** percentage of CD44^+^ and CD62L^−^ cells (T effector cells, Teff) of OT-I CD8^+^ T cells, **(C)** percentage of CD69^+^ cells of OT-I Teff cells, **(D)** median fluorescence intensity (MFI) of CD69 of OT-I Teff cells, and **(E)** percentage of interferon gamma (IFN-γ)- and TNF-α-expressing OT-I Teff cells following *in vitro* stimulation with SIINFEKL peptide (5 μM). DN, double negative for IFN-γ and TNF-α expression. Collated data from two independent experiments are shown (CTRL, *n* = 7; MAT, *n* = 10). Data in panels **(A–C)** are presented as mean + SEM. *p < 0.05, unpaired two-tailed Student’s *t*-test.

To address the fate of MAT and CTRL antigen-specific CD8^+^ T cells, we adoptively transferred ovalbumin antigen-specific CD8^+^ T cells into a host with a normal GIM prior to challenge with a systemic viral infection. Rag1KO mice do not maintain endogenous B or T cells, although subsets of innate immune cells are preserved (37), allowing for the evaluation of expansion and function of transferred ovalbumin specific OT-I T cells in response to infection. In these experiments, 1.5 × 10^5^ splenic OT-I CD8^+^ T cells isolated from day of life (dol) 15 congenic (CD45.1) MAT and CTRL infant mice were adoptively transferred into age- and gender-matched CTRL Rag1KO mice. Within 24 h, before significant homeostatic proliferation of transferred cells can occur (38), mice were infected with Vac-OVA (1 × 10^4^ PFU i.p.). At day of infection (doi) 8, which corresponds with the peak of the systemic adaptive immune response against vaccinia, we isolated lymphocytes from the site of infection in the peritoneum and from systemic draining lymphoid tissues (39). We detected equivalent percentages of MAT and CTRL OT-I CD8^+^ T cells (CD45.1^+^TCRVβ5^+^) in the PEC, MLN, and spleen of recipient mice (Figure 1A). Equivalent percentages of MAT and CTRL OT-I effector T cells (CD44^+^CD62L^−^) were detected at the site of the infection (PEC) and spleen. However, a significantly reduced percentage of MAT OT-I effectors was detected in the MLN of recipient mice (Figure 1B). Next, we analyzed the expression of the activation marker CD69 in MAT and CTRL OT-I effector T cells distributed in the spleen, MLN, and PEC of recipient mice. We detected similar percentages of CD69^+^ MAT and CTRL OT-I effector T cells at each site (Figure 1C). Nevertheless, based on the levels of expression of CD69, MAT OT-I effectors were less activated than CTRL effectors but only in the MLN (Figure 1D). Finally, we assessed the capacity of MAT and CTRL OT-I T cells isolated from the spleen, MLN, and PEC of Vac-OVA-infected CTRL Rag1KO recipient mice to produce the effector cytokines IFN-γ and TNF-α in response to the MHC class I-restricted OVA peptide SIINFEKL. Polyfunctional CD8^+^ T cell effectors are generated following vaccinia virus infection (40) and are also associated with better CTRL of viral infections (41, 42). Both MAT and CTRL OT-I effector T cells had similar percentages of IFN-γ- and TNF-α-producing cells at the site of the infection (PEC) (Figure 1E). While MAT OT-I effectors exhibited robust IFN-γ responses in peripheral lymphoid organs, CTRL OT-I effector T cells isolated in the peripheral lymphoid tissues (spleen and MLN) were characterized by a high frequency of IFN-γ and TNF-α dual producing cells (spleen 58% and MLN 61%). MAT OT-I effector T cells had a low frequency of this dual producing subset (spleen 39% and MLN 30%) and a notably higher frequency of non-cytokine producing cells (spleen 19% and MLN 20%; Figure 1E). These results illustrate that Ag-specific CD8^+^ T cells derived from MAT mice despite initial priming in a normal microbiota environment under optimal activation conditions, overall maintain a differential phenotype from CTRL Ag-specific CD8^+^ T cells in exhibiting decreased polyfunctional antiviral cytokine responses. This behavior is consistent with the hypothesis that intrinsic differences in MAT infant CD8^+^ T cells exist (36) possibly imprinted by their development in a GIM-depleted environment.

### Naïve MAT CD8^+^ T Cells Exhibit Altered TCR Signaling

We previously observed that MAT and CTRL effector CD8^+^ T cells generated *in vitro* following TCR and CD28 stimulation had a similar frequency of IFN-γ-producing cells at 24 h post-stimulation but MAT effector cells failed to sustain IFN-γ production by 72 h post-stimulation (36). TCR and CD28 engagement initiates intracellular signaling pathways that result in the activation of transcription factors that promote the activation, proliferation, differentiation, cytokine production, and survival of T cells (43). The mitogen-activated protein kinase (MAPK) and the nuclear factor-κB (NF-κB) signaling pathways are critical for T cell IFN-γ production (44–49). We hypothesized that the impaired IFN-γ production in MAT effector CD8^+^ T cells results from altered TCR-mediated signaling. Accordingly, we analyzed the expression and activation of proximal and distal proteins involved in TCR signaling, including the CD8 alpha chain (CD8α), the TCR beta chain (TCRβ), the proximal protein tyrosine kinase ZAP-70, the MAPKs Erk1/2, and the member of the NF-κB family of transcription factors, c-Rel. We surmised that intrinsic cell defects in MAT CD8^+^ T cells could occur in naïve cells or later during their effector stage. It has been described that TCR-mediated signaling responses differ between naïve, effector, and memory CD8^+^ T cell subsets (50, 51). Therefore, we enriched total T cells from the spleens of uninfected dol 15 MAT and CTRL infant mice to analyze TCR-mediated signaling in naïve and *in vitro* generated effector CD8^+^ T cells using flow cytometry.

**Figure 2:**
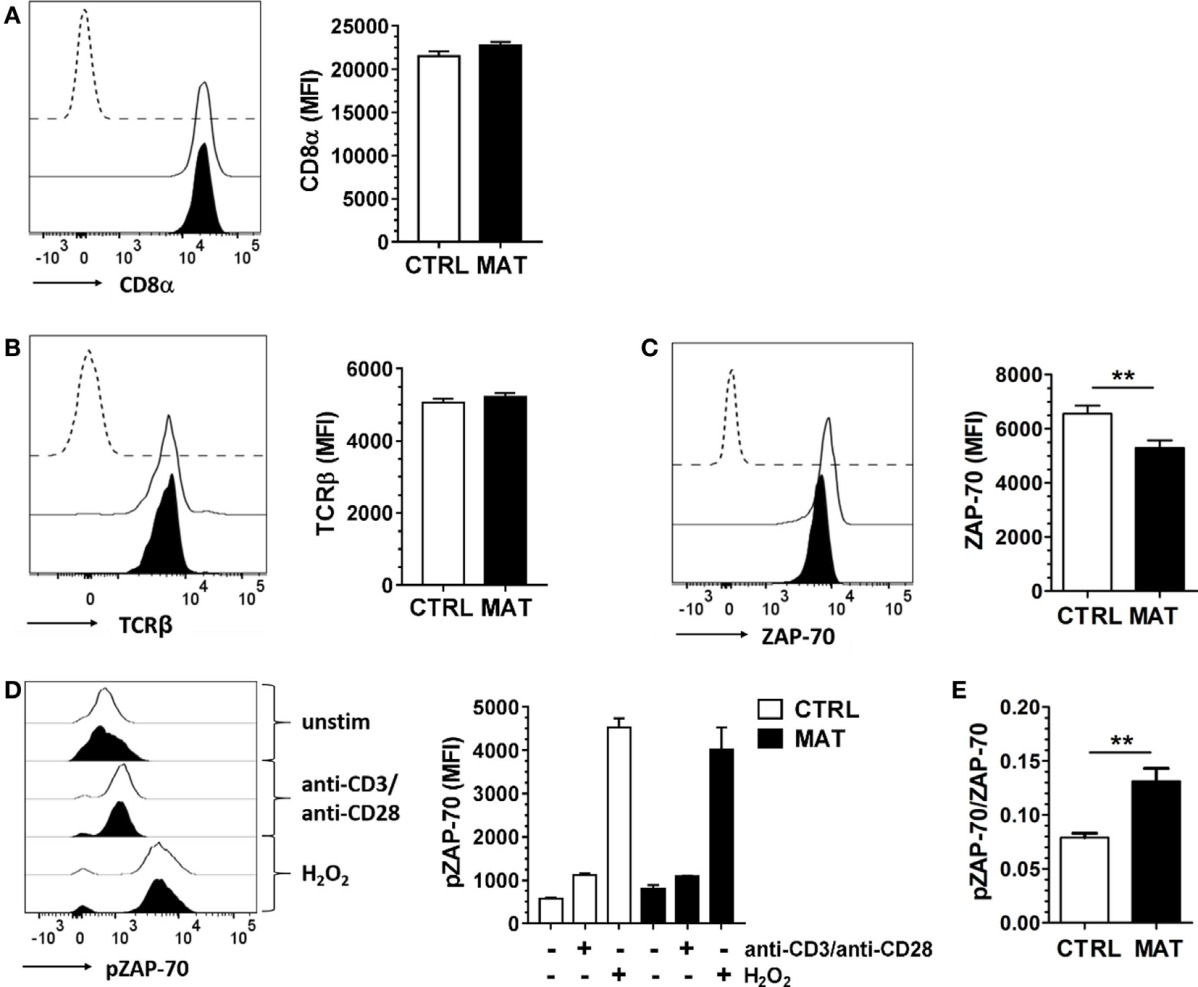
**Maternal antibiotic treatment (MAT) and control (CTRL) naïve CD8^+^ T cells display equivalent proximal T cell receptor (TCR)-mediated signaling**. Total T cells enriched from the spleens of day of life 15 CTRL and MAT infant mice were analyzed for expression of **(A)** CD8α (CTRL, *n* = 7; MAT, *n* = 5), **(B)** TCRβ (CTRL, *n* = 13; MAT, *n* = 13), **(C)** ZAP-70 (CTRL, *n* = 11, MAT, *n* = 8), and **(D)** phospho-ZAP-70 (pZAP-70) (CTRL, *n* = 6; MAT, *n* = 3) gating on naïve (CD44^−^) CD8^+^ T cells by flow cytometry. To analyze pZAP-70 expression **(D)**, the T cells were left unstimulated (−) or stimulated (+) with anti-CD3/anti-CD28 or H_2_O_2_ for 2 min at 37°C. **(E)** Ratio of pZAP-70/ZAP-70 in unstimulated naïve CD8^+^ T cells (CTRL, *n* = 6; MAT, *n* = 3). Representative graphs (CTRL, white; MAT, black; dashed line, negative staining control) and analysis of the median fluorescence intensity (MFI) are shown. Data are representative of two independent experiments and presented as mean + SEM. **p < 0.01, unpaired two-tailed Student’s *t*-test.

First, we analyzed CD8α and TCRβ expression in MAT and CTRL naïve CD8^+^ T cells. We noted equivalent expression of these cell surface proteins in both groups (Figures 2A,B). Next, we analyzed the expression of the proximal TCR signaling molecule ZAP-70. Despite reduced protein expression levels in MAT naïve CD8^+^ T cells (Figure 2C), both MAT and CTRL cells exhibited equivalent expression levels of pZAP-70 following TCR and CD28 crosslinking (Figure 2D). Moreover, H_2_O_2_ stimulation, which bypasses TCR stimulation and induces ZAP-70 tyrosine phosphorylation (52), resulted in equivalent activation of ZAP-70 in MAT and CTRL naïve CD8^+^ T cells (Figure 2D). However, unstimulated MAT naïve CD8^+^ T cells exhibited an increased pZAP-70/ZAP-70 ratio compared with CTRLs (Figure 2E). Collectively, these results indicated that proximal TCR-mediated signaling capacity is intact in MAT naïve CD8^+^ T cells and that ZAP-70 may be hyper-phosphorylated in these cells.

**Figure 3:**
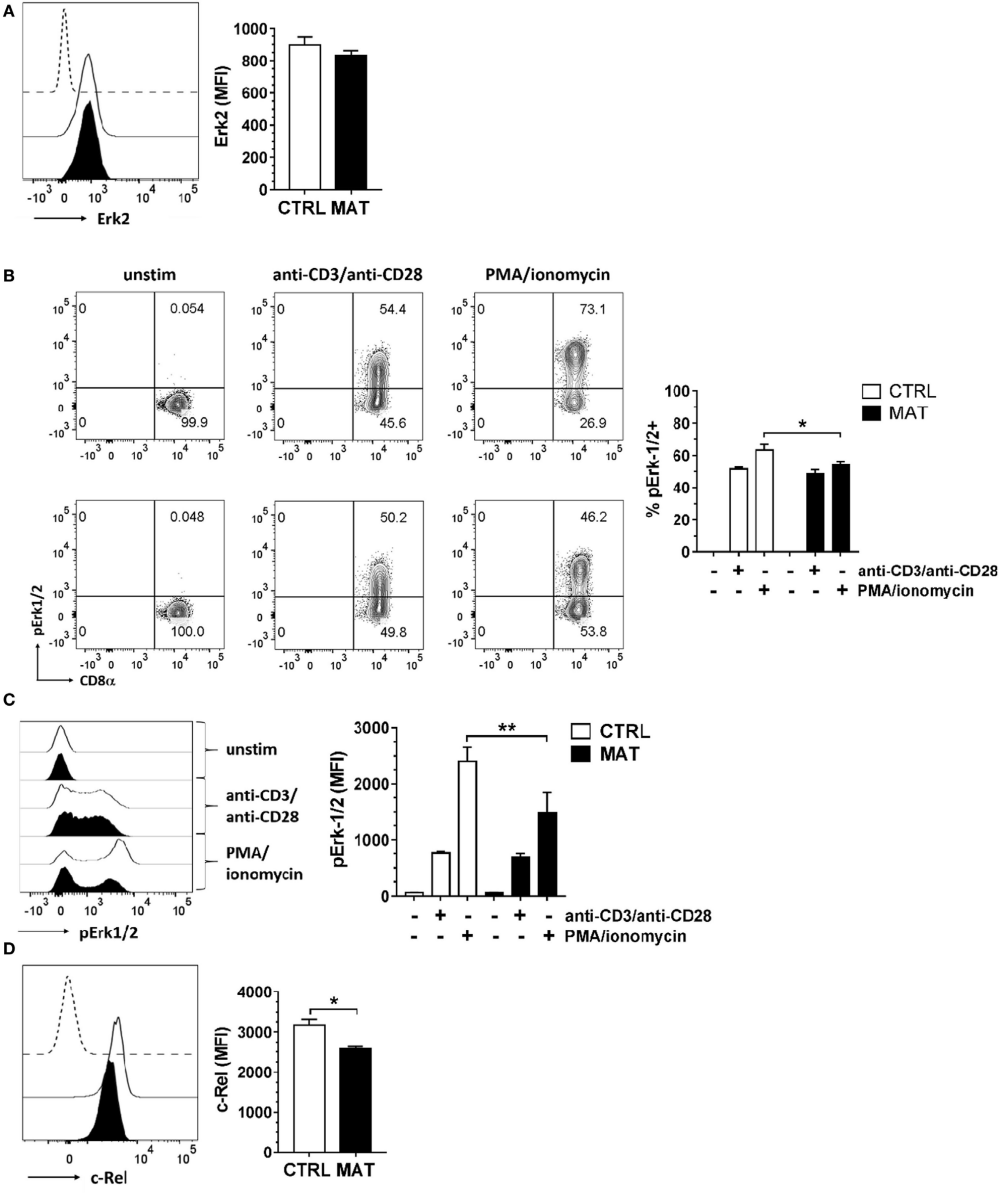
**Distal T cell receptor-mediated signaling is compromised in maternal antibiotic treatment (MAT) naïve CD8^+^ T cells**. Total T cells enriched from the spleens of day of life 15 control (CTRL) and MAT infant mice were analyzed for **(A)** expression of Erk2 (CTRL, *n* = 5; MAT, *n* = 4), **(B)** percentage of phospho-Erk-1/2 (pErk1/2) positive cells (CTRL, *n* = 5; MAT, *n* = 4), **(C)** expression of pErk1/2 (CTRL, *n* = 5; MAT, *n* = 4), and **(D)** expression of c-Rel (CTRL, *n* = 6; MAT, *n* = 3) gating on naïve (CD44^−^) CD8^+^ T cells by flow cytometry. To analyze pErk1/2 expression **(B,C)**, T cells were left unstimulated (−) or stimulated (+) with anti-CD3/anti-CD28 or phorbol 12-myristate 13-acetate (PMA)/ionomycin for 2 min at 37°C. Representative graphs (CTRL, white; MAT, black; dashed line, negative staining control) and analysis of the median fluorescence intensity (MFI) or percentage are shown. Data are representative of two independent experiments, presented as mean + SEM and were analyzed by one-way ANOVA with Holm–Sidak posttest **(B,C)** or unpaired two-tailed Student’s *t*-test **(D)**. *p < 0.05, **p < 0.01.

We then evaluated distal signaling components of the TCR signaling pathway, specifically focusing on the MAPK pathway regulated by Erk1/2, since its activation is critical for T cell activation and differentiation (53). MAT and CTRL naïve CD8^+^ T cells displayed similar expression levels of Erk2 (Figure 3A) and following TCR/CD28 crosslinking, equal percentages (Figure 3B) and expression levels of pErk1/2 (Figure 3C). In order to determine if protein kinase C (PKC) signaling upstream of the Erk1/2 MAPK pathway was equivalent in MAT and CTRL naïve CD8^+^ T cells, we bypassed TCR signaling by activating T cells with a combination of the phorbol ester PMA and the calcium ionophore ionomycin. PMA induces PKC auto-phosphorylation by mimicking the natural ligand diacylglycerol and ionomycin synergizes with PMA (54). We determined that MAT naïve CD8^+^ T cells had reduced frequency and expression levels of pErk1/2 upon PMA and ionomycin stimulation, suggesting that the activation of PKC and Ca^2+^ mobilization may be somewhat compromised in these cells (Figures 3B,C). Finally, we analyzed the expression of c-Rel, a component of the NF-κB signal transduction pathway, in MAT and CTRL naïve CD8^+^ T cells. Its expression levels were significantly reduced in MAT cells compared with CTRLs (Figure 3D). Collectively, our results demonstrate that although MAT and CTRL naïve CD8^+^ T cells display equivalent proximal TCR-mediated signaling, distal- and calcium-dependent signaling events could be compromised.

### Effector MAT CD8^+^ T Cells Do Not Sustain Erk1/2 Phosphorylation

**Figure 4:**
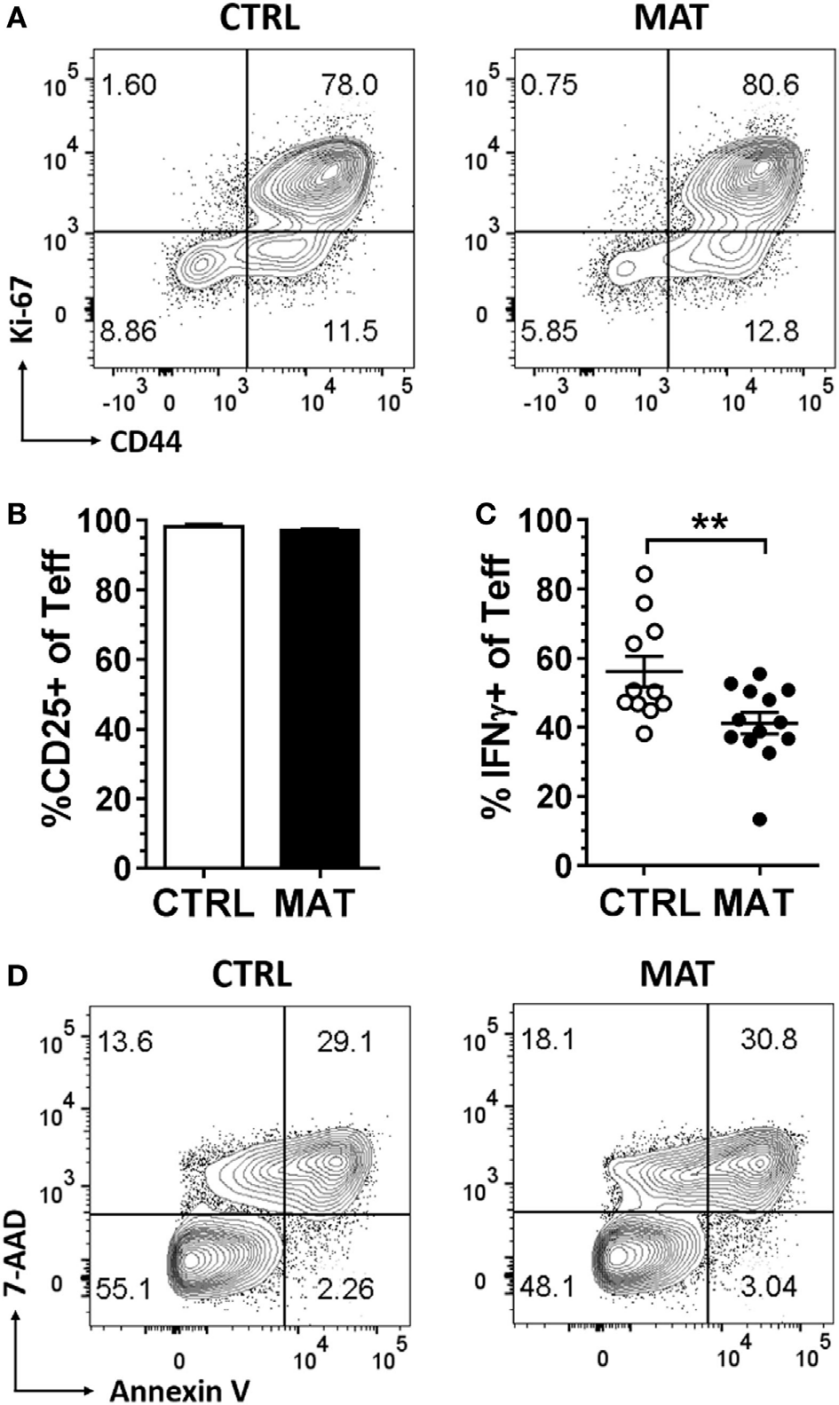
**Maternal antibiotic treatment (MAT) and control (CTRL) effector CD8^+^ T cells proliferate and activate equally, but MAT effectors produce less interferon gamma (IFN-γ)**. CD8^+^ T cells purified from pooled spleens of day of life 15 CTRL and MAT infant mice were stimulated with anti-CD3/anti-CD28 for 72 h and analyzed for **(A)** percentage of Ki-67 and CD44^+^ cells, **(B)** percentage of CD25^+^ cells of T effector cells (CD44^+^CD62L^−^, Teff) (CTRL, *n* = 6; MAT, *n* = 10), **(C)** percentage of IFN-γ^+^ Teff cells (CTRL, *n* = 11; MAT, *n* = 13), and **(D)** percentage of 7-aminoactinomycin D and annexin V positive Teff cells by flow cytometry. Data are representative of three independent experiments, presented as mean + SEM and were analyzed by unpaired two-tailed Student’s *t*-test **(B,C)**. **p < 0.01.

In order to generate effector CD8^+^ T cells, we purified CD8^+^ T cells from gender-matched pooled spleens of dol 15 CTRL and MAT infant mice, and stimulated CD8^+^ T cells with anti-CD3 and anti-CD28 for up to 72 h. At this point, the CD8^+^ T cells acquired an effector status based on the expression of activation and differentiation cell surface markers, proliferation, and cytokine production (Figure 4). Both MAT and CTRL CD8^+^ T cells exhibited equivalent proliferation and differentiation based on the expression of Ki-67 and CD44 (Figure 4A). However, despite identical frequencies of CD25^+^ effector cells (Figure 4B), a significantly reduced percentage of MAT effector cells produced IFN-γ in comparison with CTRL effectors (Figure 4C), consistent with our previous findings. This was not related to differences in cell survival since both MAT and CTRL effector CD8^+^ T cells exhibited identical percentages of annexin V and 7-AAD positive cells (Figure 4D).

**Figure 5:**
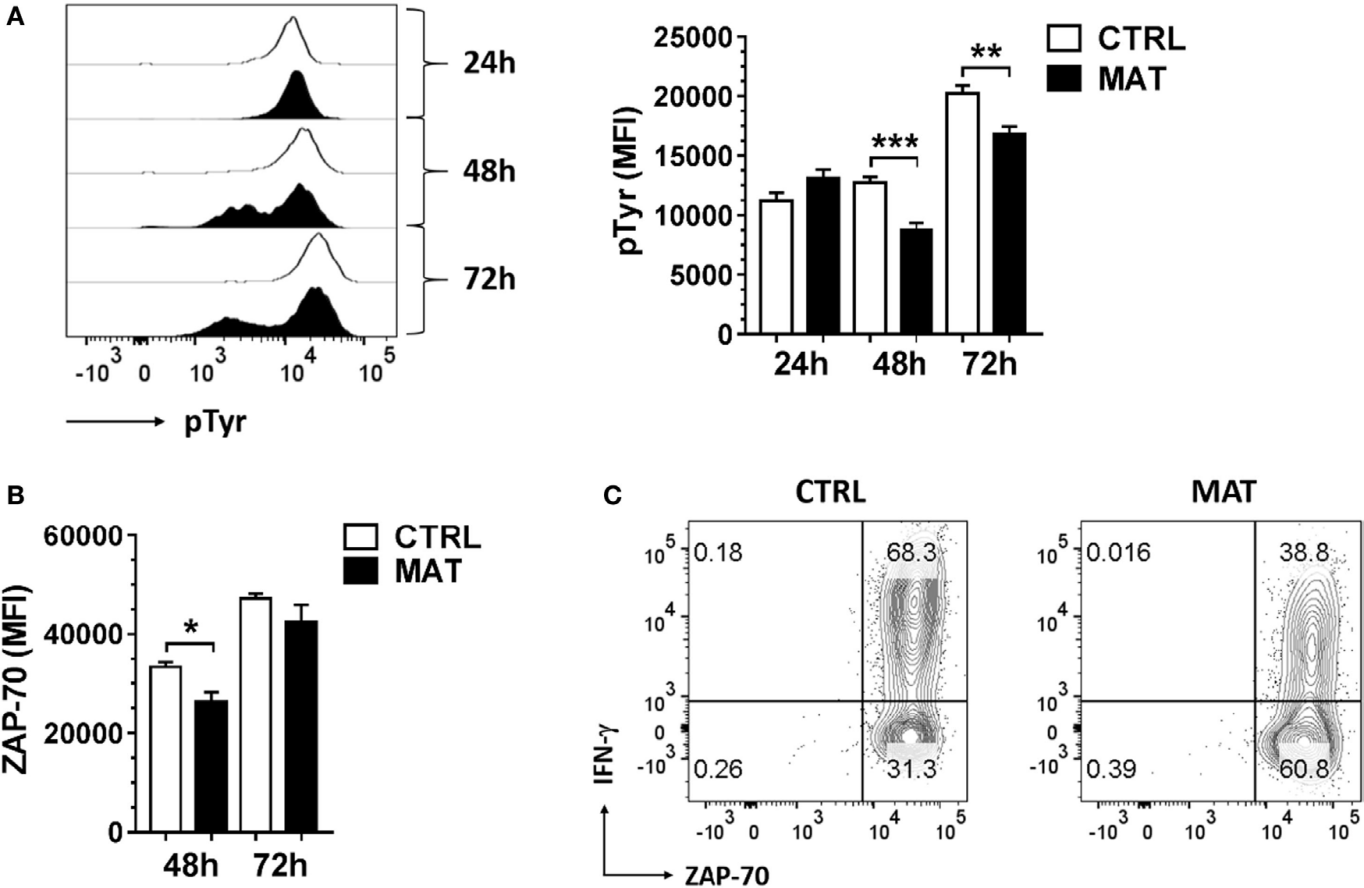
**Maternal antibiotic treatment (MAT) effector CD8^+^ T cells do not sustain total protein tyrosine phosphorylation**. Total T cells enriched from the spleens of day of life 15 control (CTRL) and MAT infant mice were stimulated with anti-CD3/anti-CD28 for 24, 48, and 72 h and analyzed for **(A)** expression of total protein tyrosine phosphorylation (pTyr) (CTRL, *n* = 6; MAT, *n* = 5), **(B)** expression of ZAP-70 (CTRL, *n* = 7; MAT, *n* = 6), and **(C)** percentage of ZAP-70 and interferon gamma (IFN-γ) positive cells gating on effector (CD44^+^) CD8^+^ T cells by flow cytometry. Representative graphs (CTRL, white; MAT, black) and analysis of the median fluorescence intensity (MFI) are shown. Data are representative of two independent experiments and presented as mean + SEM. *p < 0.05, **p < 0.01, ***p < 0.001, one-way ANOVA with Holm–Sidak posttest.

Thus, in order to identify at which time point during effector T cell differentiation, MAT CD8^+^ T cells are impaired in their capacity to efficiently transduce signals through the TCR to sustain IFN-γ production, we analyzed total protein tyrosine phosphorylation (pTyr), ZAP-70 expression, and expression/activation of Erk-1/2 at 24, 48, and 72 h post-stimulation. In these assays, we enriched total T cells from the spleens of dol 15 CTRL and MAT infant mice, and stimulated T cells with anti-CD3 and anti-CD28 for the indicated time points. Analysis was then performed gating on effector (CD44^+^) CD8^+^ T cells. The TCR-induced total protein tyrosine phosphorylation analysis revealed that MAT and CTRL effector CD8^+^ T cells express similar levels of tyrosine phosphorylated proteins at 24 h post-stimulation. However, over the course of the subsequent 48 h, these levels were markedly reduced in MAT effector CD8^+^ T cells in comparison with CTRL effectors, indicating that TCR-mediated signaling events in MAT CD8^+^ T cells are not sustained during the later effector stages (Figure 5A).

ZAP-70 expression in T cells increases following sustained antigenic stimulation, and elevated ZAP-70 protein levels in activated CD4^+^ T cells have been associated with acquisition of effector functions (i.e., IFN-γ production) (55). Therefore, we analyzed ZAP-70 expression in MAT and CTRL effector CD8^+^ cells at 48 and 72 h post-stimulation. Although ZAP-70 expression was reduced in MAT effector CD8^+^ T cells at 48 h post-stimulation, its expression was equivalently upregulated in both MAT and CTRL effector CD8^+^ T cells by 72 h (Figure 5B). Hence, reduced IFN-γ production in MAT effector CD8^+^ T cells at 72 h post-stimulation was not related to impaired ZAP-70 expression (Figure 5C).

**Figure 6:**
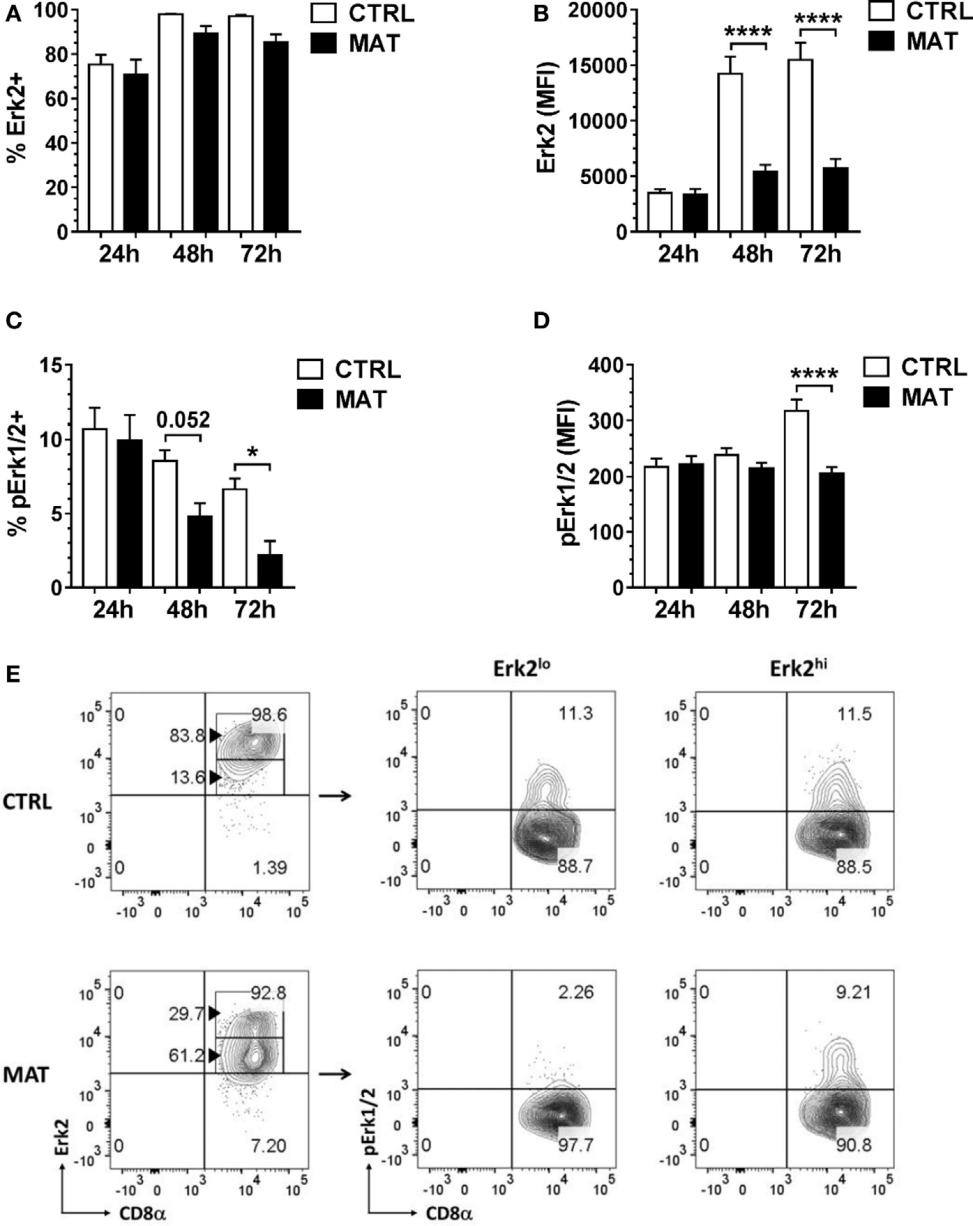
**Maternal antibiotic treatment (MAT) effector CD8^+^ T cells do not sustain phosphorylation of Erk-1/2**. Total T cells enriched from the spleens of day of life 15 control (CTRL) and MAT infant mice were stimulated with anti-CD3/anti-CD28 for 24, 48, and 72 h and analyzed for **(A)** percentage of Erk2^+^ cells (CTRL, *n* = 7; MAT, *n* = 6), **(B)** expression of Erk2 (CTRL, *n* = 7; MAT, *n* = 6), **(C)** percentage of phospho-Erk-1/2 (pErk1/2) positive cells (CTRL, *n* = 7; MAT, *n* = 6), and **(D)** expression of pErk1/2 gating on effector (CD44^+^) CD8^+^ T cells (Teff). **(E)** Percentage of CD8^+^ Teff cells expressing low or high levels of Erk2 (Erk2^lo^ and Erk2^hi^, respectively) and gating analysis of their corresponding pErk1/2-expressing populations at 48 h post-stimulation. Data are representative of two independent experiments and presented as mean + SEM. *p < 0.05, ****p < 0.0001, one-way ANOVA with Holm–Sidak posttest.

Given the similarity in ZAP-70 expression, we examined distal signaling molecules required for cytokine gene transcription in effector T cells. We focused our analysis on the Erk-1/2 signaling pathway and Erk2 given its role in T cell activation and IFN-γ transcription (46, 47). At 24 h post-stimulation, we detected similar frequency and expression levels of Erk2 in MAT and CTRL effector CD8^+^ T cells (Figures 6A,B). In addition, we determined that MAT and CTRL effector CD8^+^ T cells displayed equivalent percentages and expression levels of pErk1/2 following TCR and CD28 crosslinking (Figures 6C,D). However, as early as 48 and 2 h post-stimulation, MAT effector CD8^+^ T cells displayed significantly reduced frequency and expression levels of pErk1/2 compared with CTRL effector cells (Figures 6C,D). Although the percentage of Erk2-expressing MAT and CTRL CD8^+^ T cells remained equivalent at 48 and 72 h post-stimulation (Figure 6A), MAT effector cells expressed significantly lower amounts of Erk2 at these time points (Figure 6B). Closer analysis revealed that this was due to bimodal expression of Erk2 in MAT effector cells. As shown in Figure 6E, the majority of MAT effector CD8^+^ T cells expressed intermediate to low levels of Erk2 whereas most CTRL effectors expressed high levels of this protein at 48 h post-stimulation. Moreover, while both Erk2^lo^- and Erk2^hi^-expressing CTRL effector CD8^+^ T cells had phosphorylated Erk-1/2, only Erk2^hi^-expressing MAT effector CD8^+^ T cells had phosphorylated Erk-1/2 at 48 h post-stimulation (Figure 6E). Furthermore, by 72 h post-stimulation, both Erk2^lo^- and Erk^hi^-expressing MAT effector CD8^+^ T cells lacked phosphorylated Erk-1/2 while CTRL effector CD8^+^ T cells retained Erk2 expression and sustained phosphorylation of Erk-1/2 (Figure S1 in Supplementary Material). Moreover, we observed the exact Erk2 and pErk1/2 expression pattern in MAT effector CD8^+^ T cells generated upon stimulation of purified CD8^+^ T cells with anti-CD3 and anti-CD28 for 72 h (Figure S2 in Supplementary Material). This result suggests a differentially regulated control of signaling responsiveness (56) in MAT effector CD8^+^ T cells that may contribute to the lack of sustained activation of Erk-1/2 (57).

### LPS Treatment Enhances IFN-γ Production in MAT Effector CD8^+^ Cells *In Vivo* and *In Vitro*

**Figure 7:**
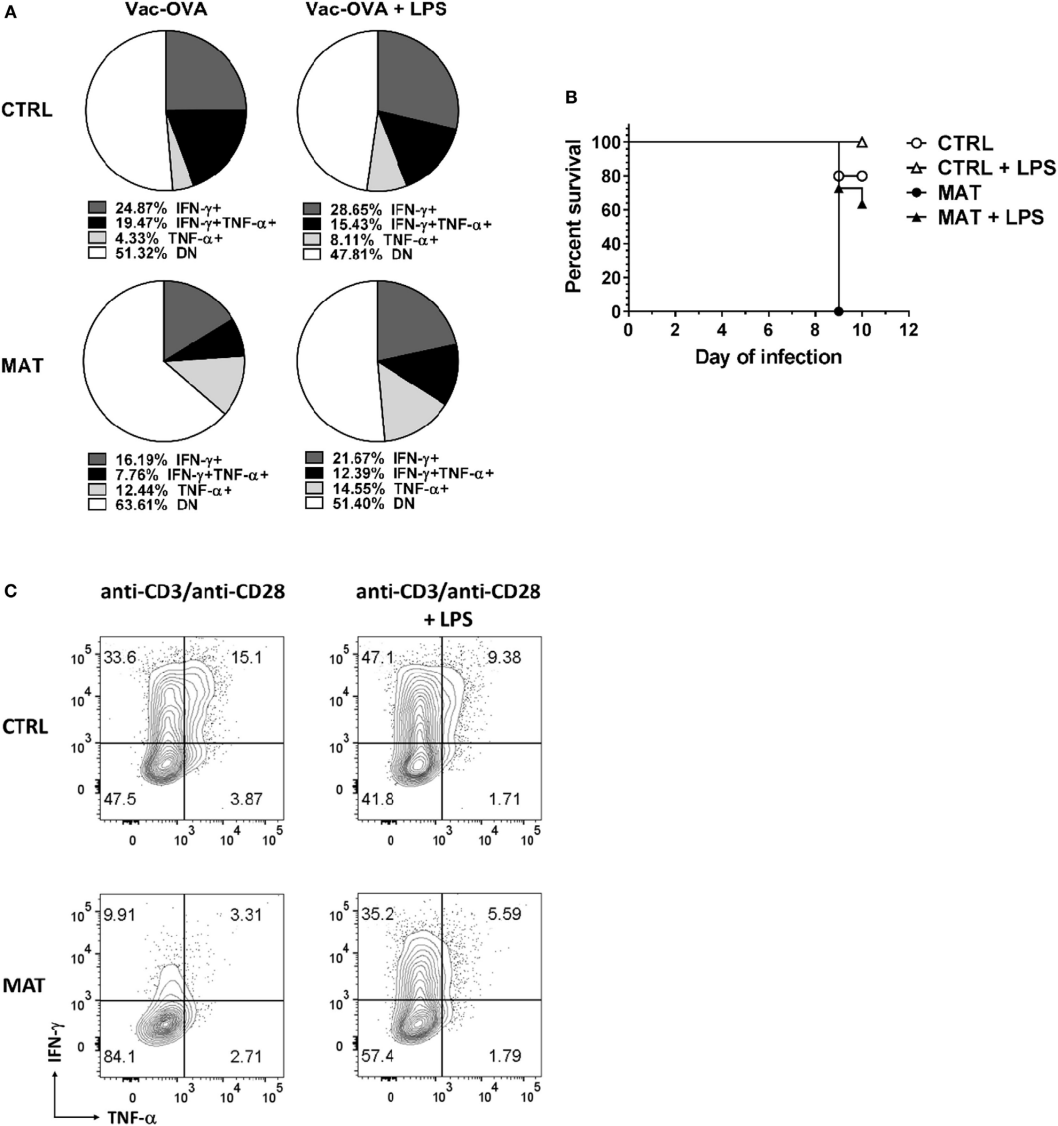
**Lipopolysaccharide (LPS) treatment enhances interferon gamma (IFN-γ) production in maternal antibiotic treatment (MAT) effector CD8^+^ cells *in vivo* and *in vitro***. Control (CTRL) and MAT infant mice were infected at day of life (dol) 15 with vaccinia-ovalbumin (Vac-OVA, 1 × 10^4^ PFU intraperitoneal) and were treated with *Escherichia coli*-derived LPS (50 μg o.g.) every other day for 10 days (Vac-OVA + LPS). **(A)** At day of infection 11, lymphocytes were isolated from the spleens of CTRL and MAT mice infant mice and stimulated *in vitro* with SIINFEKL peptide, phorbol 12-myristate 13-acetate, and ionomycin for 5 h to analyze the percentage of IFN-γ- and TNF-α-producing CD8^+^ T effector cells (CD44^+^CD62L^−^, Teff) by flow cytometry (CTRL Vac-OVA, *n* = 3; CTRL Vac-OVA + LPS, *n* = 7; MAT Vac-OVA, *n* = 2; MAT Vac-OVA + LPS, *n* = 3). **(B)** Survival curve of CTRL and MAT infant mice following Vac-OVA infection and LPS treatment (CTRL Vac-OVA, *n* = 5; CTRL Vac-OVA + LPS, *n* = 5; MAT Vac-OVA, *n* = 2; MAT Vac-OVA + LPS, *n* = 11). Comparison of survival curves was performed by log-rank (Mantel–Cox) test. Data are representative of two infection experiments. **(C)** Pooled CD8^+^ T cells isolated from the spleens of uninfected dol 15 CTRL (*n* = 3) and MAT (*n* = 6) infant mice were stimulated with anti-CD3/anti-CD28 with or without *Escherichia coli*-derived LPS (1 μg/ml) for 72 h and analyzed for the percentage of IFN-γ and TNF-α-producing Teff cells by flow cytometry. Data are representative of three independent experiments.

**Figure 8:**
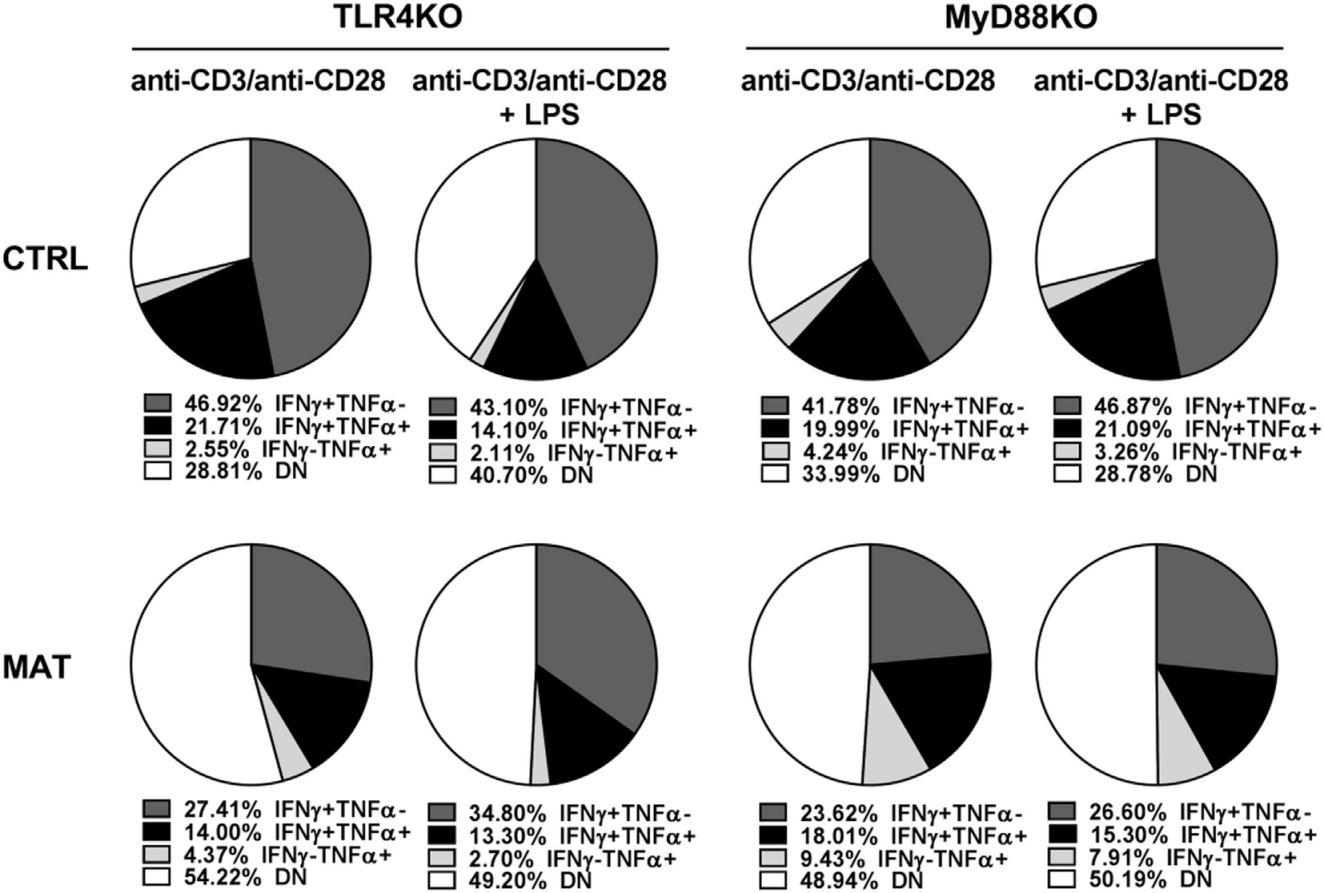
**Cytokine production of control (CTRL) and maternal antibiotic treatment (MAT) TLR4KO and MyD88KO effector CD8^+^ T cells in response to lipopolysaccharide (LPS)**. Pooled CD8^+^ T cells isolated from the spleens of uninfected day of life 15 CTRL TLR4KO (*n* = 2), MAT TLR4KO (*n* = 5), CTRL MyD88KO (*n* = 5), and MAT MyD88KO (*n* = 4) infant mice were stimulated with anti-CD3 and anti-CD28 with or without *Escherichia coli*-derived LPS (1 μg/ml) for 72 h. Analysis of the percentage of interferon gamma (IFN-γ) and TNF-α-producing effector CD8^+^ T cells (CD44^+^CD62L^−^) was performed by flow cytometry. DN, double negative for IFN-γ and TNF-α expression. Data are representative of two independent experiments.

It was shown that LPS treatment could restore impaired T cell IFN-γ production in an antibiotic-mediated GIM dysbiosis adult mouse model of influenza A virus infection (58). Thus, we decided to evaluate if LPS treatment *in vivo*, as a proxy for colonization by Gram-negative bacteria that are missing in MAT mice (36), could enhance T cell-mediated cytokine production and rescue MAT infant mice from dying following systemic viral infection. Dol 15 MAT and CTRL infant mice were infected with Vac-OVA (1 × 10^4^ PFU i.p.) and treated with *E. coli*-derived LPS (50 μg o.g.) beginning on the day of the infection and continuing every other day for 10 days. At doi 11, we analyzed the cytokine response of splenic MAT and CTRL effector CD8^+^ T cells and observed that LPS treatment enhanced IFN-γ and TNF-α production in MAT effector CD8^+^ T cells to CTRL levels whereas it had a modest effect in CTRL cells (Figure 7A). Moreover, LPS treatment significantly increased the survival of MAT infants, while untreated animals succumbed at the peak of the viral infection, 60% of LPS-treated MAT mice survived systemic vaccinia virus infection (Figure 7B). We also evaluated the effect of LPS treatment on IFN-γ and TNF-α production from MAT splenic effector CD8^+^ T cells generated *in vitro*. MACS-enriched splenic CD8^+^ T cells were stimulated with anti-CD3/CD28 in the absence or presence of *E. coli* 055:B5-derived LPS (1 μg/ml) for 72 h. The purity of CD8^+^ T cells in these cultures was up to 97% (Figure S3 in Supplementary Material). Compared with CTRL effector CD8^+^ T cells, MAT effectors cultured in the absence of LPS again demonstrated a reduced percentage of IFN-γ- and TNF-α-producing cells. Including LPS during stimulation of MAT spleen-derived effectors resulted in enhanced IFN-γ production and unchanged TNF-α production (Figure 7C). The effect of the LPS seemed to be specific as we also performed the same assays using CD8^+^ T cells purified from gender-matched pooled spleens of dol 15 MAT and CTRL TLR4KO and MyD88KO infant mice. We observed that TLR4KO and MyD88KO MAT effectors stimulated in the absence of LPS exhibited reduced percentages of IFN-γ- and TNF-α-producing cells than CTRLs, and the addition of LPS during TCR stimulation did not enhance cytokine production profiles in either CTRL or MAT-derived CD8^+^ T cells (Figure 8). These results suggest that in certain circumstances of GIM dysbiosis, LPS treatment could represent an effective intervention to restore CD8^+^ T cell function and enhance resistance against viral infections.

## Discussion

A goal of our research is to understand how the gastrointestinal microbiome (GIM) influences adaptive antiviral immunity during infancy. We have discovered that CD8^+^ T cells from infants born of mothers treated with antibiotics (MAT) exhibit markedly diminished IFN-γ responses in contrast to age-matched CTRL mice developing with a normal GIM. MAT CD8^+^ T cell responses were depressed both *in vivo* following sublethal viral infection and *in vitro* following TCR/CD28 stimulation. The *in vitro* experiments indicated that MAT infant CD8^+^ T cells maintain intrinsic cell dysfunction when removed from their host environment. This intrinsic dysfunction was not fully restored following transfer of 15-day-old MAT T cells into an age-matched non-dysbiotic host environment or by treatment with *E. coli*-derived LPS. Collectively, our results demonstrate that systemic T cells that develop in infant mice with GIM dysbiosis exhibit T cell dysfunction due to altered expression and activation of key TCR signaling proteins thus compromising sustained T cell cytokine production.

Our results suggest that the dysbiotic environment present in infant MAT mice imprints their peripheral CD8^+^ T cells and suppresses intrinsic responsiveness, although the exact mechanism mediating this effect is not yet clear. While infant T cells can generate adult-like responses in specific circumstances (59), emerging studies of neonatal T cells from a normal host microbiota environment support that intrinsic properties distinguish their TCR responsiveness from juvenile and adult T cells (60, 61). Human cord blood-derived CD4^+^ T cells were found deficient in TCR-associated signaling molecules including low Lck expression, inefficient phosphorylation of Lck, and downstream reduced TCR-associated protein expression (62, 63). Additionally, cord blood-derived human neonatal CD8^+^ T cells appear to require IL-12 to provide a “third signal” following CD3 and CD28 stimulation in order to achieve maximal expansion, proliferation, IL-2 production, and differentiation into IFN-γ producing effector cells (64). In order to achieve the maximal phosphorylation of proximal signaling and later stage TCR signaling molecules, sustained IL-12 signaling is required for at least 72 h. Thus, proximal and distal signaling events may not act in concert in infant T cells modifying their responsiveness to TCR-dependent signaling for activation and differentiation. We similarly noted differences in the proximal expression and downstream sustained phosphorylation in MAT CD8^+^ T cells. The finding of differential expression of Erk1/2 in MAT effector CD8^+^ T cells was surprising yet the lower expression of Erk1/2 correlated with diminished and poorly sustained activation in these cells. We speculate that this differential expression could be due to alternative splice variants or other intrinsic factors regulating posttranslational protein stability of Erk1/2 (65) in MAT effector CD8^+^ T cells. Future experiments will be directed to evaluate these possibilities. Overall, our results indicate a requirement for a numerically dense and diverse GIM in contributing to full effector function of infant CD8^+^ T cells.

In addition to T cell-signaling differences, infants are also characterized by a distinct GIM (66). Therefore, the functional behavior and phenotype of infant adaptive T cell responses regulated by the GIM may not be equivalent to adults. In adult mice, depleting the microbiota by antibiotics suppressed inflammasome activation and expression of antiviral defense genes in innate immune cells resulting in poor influenza-specific or LCMV-specific T cell responses (67, 68). However, our results indicate that dysbiosis of the infant GIM directly impacts infant CD8^+^ T cells further compromising the already reduced capacity of infant T cells to generate sufficient IFN-γ driven responses (28).

We observed an increased pZAP-70/ZAP-70 ratio in unstimulated MAT naïve CD8^+^ T cells with respect to CTRL cells. There is precedence that an altered GIM can effect baseline activation of systemic T cells. Huang et al. established a restricted flora mouse model in which mice are maintained with a limited microbiota of defined by six non-pathogenic *Clostridium* species. They observed that adult splenic naïve CD4^+^ T cells exhibited TCR hyperresponsiveness that was associated with increased phosphorylation of the signaling molecules ZAP-70, Lck, and LAT, and increased activation induced cell death (69). It is possible that alterations in TCR-mediated signaling in MAT T cells include hyper-phosphorylated basal levels of critical proximal and intermediate signaling proteins in order to compensate for downstream deficiencies. Just as Huang et al., we similarly speculate that the reduced microbial environment in MAT mice either suppresses a baseline state required for T cell functional homeostasis or that circulating metabolites of the microbiome regulate differential responsiveness of CD8^+^ T cells. A reduction in the density or diversity of species producing immune regulating metabolites could thus impact normal patterns of transcriptional and signal regulation in peripheral infant T cells dependent upon these products.

The myriad of/and mechanisms by which microbiota-derived products mediate immune effects still remains to be fully characterized (70, 71). In a recent examination, the capacity of inflammatory cytokine production in human peripheral blood lymphocytes has been linked to the GIM and specific microbial metabolic pathways (72). In addition, two well described examples of microbial-derived factors are shown to regulate T cells *in vivo*. PSA produced by *Bacteroides fragilis* engages TLR2 expressed on T cells to promote Treg differentiation in peripheral lymphoid tissues (73). The short-chain fatty acid butyrate serves as an energy source for colonic epithelial cells but also mediates immune system homeostasis and function by binding to GPR43 expressed on a variety of innate and adaptive immune cells, specifically Tregs (74). Butyrate is also able to direct epigenetic gene modification by acting on HDAC (35). Given the importance of epigenetic mechanisms involved in regulating development and function of infant T cells (32, 75), it is interesting to speculate regarding their permissiveness to circulating bacterial metabolites derived from the infant GIM. A direct effect of LPS on enriched or purified human and murine T cells *in vitro* has been previously described (76). However, we cannot completely rule out that the *in vitro* effects of LPS CD8^+^ T cells in our assay were mediated *via* contaminating innate effectors since we did not use sort purified cells. Nonetheless, LPS has been successfully used as an adjuvant to enhance viral- and vaccine-specific responses *in vivo* (58, 77), and we did observe a positive effect of bacterial-derived LPS on enhancing MAT T cell function and MAT infant mouse survival following viral infection. The partial response observed in the mice could indicate that treatment occurred too late to fully rescue MAT functional responses and that specific age windows exist when a normal GIM must be present to support the development and function of normal immunity.

Infancy and childhood is a period marked by enhanced baseline susceptibility to viral infections and is also the period of life when the majority of vaccines are given. Thus, an evaluation of how GIM dysbiosis during this period of life can alter T cell immunity is critical for our understanding of infant immunity in general, and for developing strategies that can protect or enhance CD8^+^ T cell immunity. Our results suggest that the infant GIM imprints CD8^+^ T cell function during this period of life and could have long-term consequences. Our ongoing and future analysis of this model includes (1) determining the long-lasting impact of GIM dysbiosis on T cell function, particularly in the generation of long-term memory, (2) testing strategies that can be used to correct the dysfunction, (3) and evaluating the role of infant-derived GIM microbial metabolites in regulating CD8^+^ T cell antiviral immunity.

## Author Contributions

GG-P and EL-S designed and performed experiments, analyzed and interpreted data, prepared figures, and wrote the manuscript.

## Conflict of Interest Statement

The authors declare that the research was conducted in the absence of any commercial or financial relationships that could be construed as a potential conflict of interest.

## Abbreviations

GIM, gastrointestinal microbiome; TCR, T cell receptor; ERK, extracellular signal-regulated kinase; LPS, lipopolysaccharide; TLR, toll-like receptor; PMA, phorbol 12-myristate 13-acetate.
