# Supplementary material for: Gastrointestinal Microbiome Dysbiosis in Infant Mice Alters Peripheral CD8+ T Cell Receptor Signaling
Source: Front Immunol. 2017 Mar 8;8:265. doi: 10.3389/fimmu.2017.00265 (PMC5340779; doi:10.3389/fimmu.2017.00265)
Supplement: Supplementary file 1 [file Presentation_1.pdf]

Supplementary Material

Gastrointestinal microbiome dysbiosis in infant mice alters peripheral $CD8^+$ T cell receptor signaling

Authors: Gabriela Gonzalez-Perez[1] and Esi S.N. Lamousé-Smith[1*],
[1]Division of Pediatric Gastroenterology, Hepatology and Nutrition, Department of Pediatrics, Columbia University Medical Center, New York, NY 10032

***Correspondence**: Corresponding Author enl2118@cumc.columbia.edu

Supplementary Figure 1

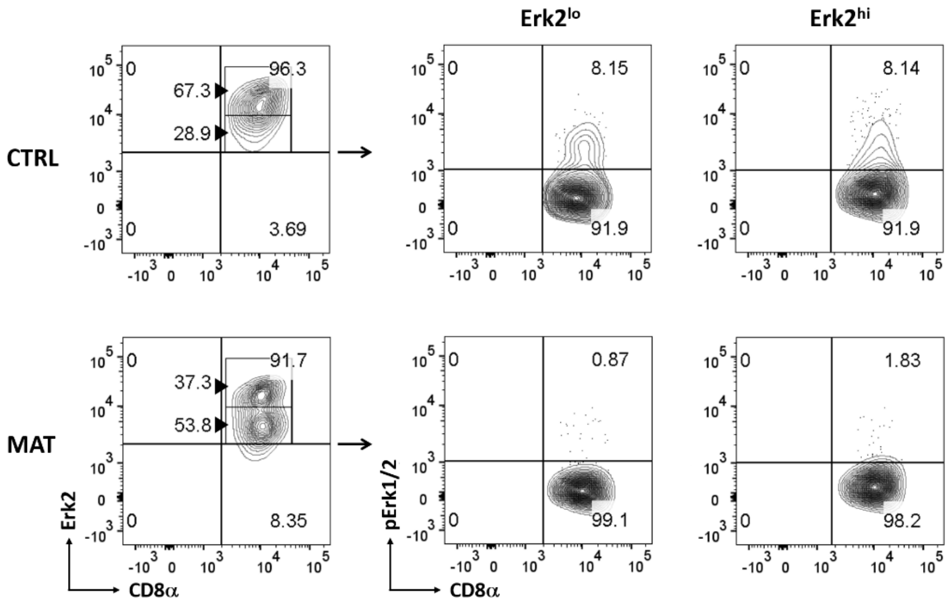

Supplementary Figure 1. MAT effector $CD8^+$ T cells do not sustain phosphorylation of Erk-1/2. Total T cells enriched from the spleens of uninfected dol 15 CTRL and MAT infant mice were stimulated with anti-CD3/anti-CD28 for 72 hours and effector ($CD44^+$) $CD8^+$ T cells were analyzed for the expression of Erk2 and phospho-Erk-1/2 (pErk-1/2) by flow cytometry. The percentage of effector $CD8^+$ T cells expressing low or high levels of Erk2 ($Erk2^{lo}$ and $Erk2^{hi}$, respectively) and the gating analysis of their corresponding pErk-1/2 expressing populations is shown. Data is representative of two independent experiments.

Supplementary Figure 2

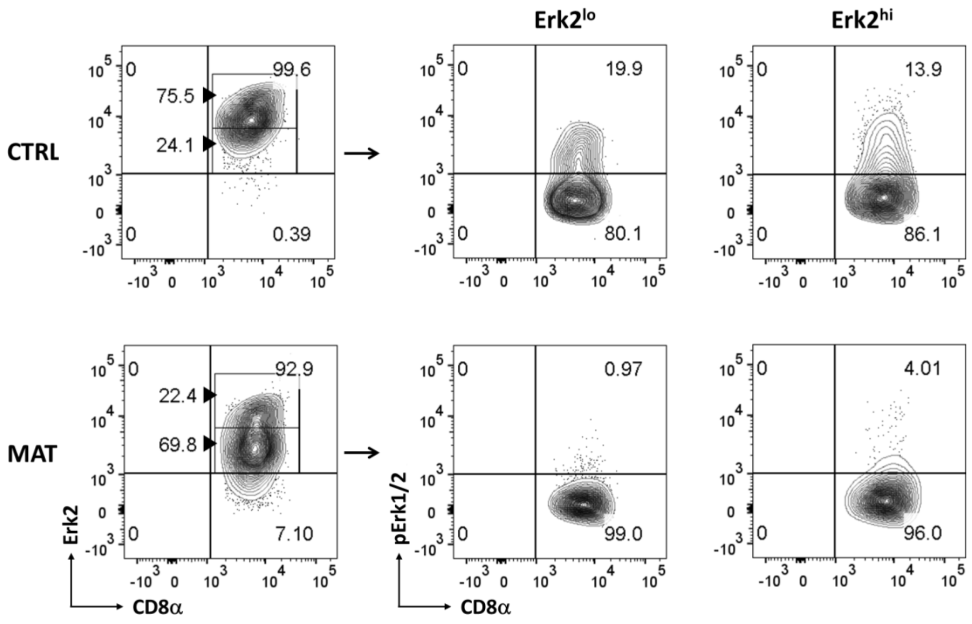

Supplementary Figure 2. Purified MAT effector CD8$^+$ T cells do not sustain phosphorylation of Erk-1/2. Total CD8$^+$ T cells purified from gender-matched pooled spleens of uninfected dol 15 CTRL and MAT infant mice were stimulated with anti-CD3/anti-CD28 for 72 hours and effector (CD44$^+$) CD8$^+$ T cells were analyzed for the expression of Erk2 and phospho-Erk-1/2 (pErk-1/2) by flow cytometry. The percentage of effector CD8$^+$ T cells expressing low or high levels of Erk2 (Erk2^{lo} and Erk2^{hi}, respectively) and the gating analysis of their corresponding pErk-1/2 expressing populations is shown.

Supplementary Figure 3

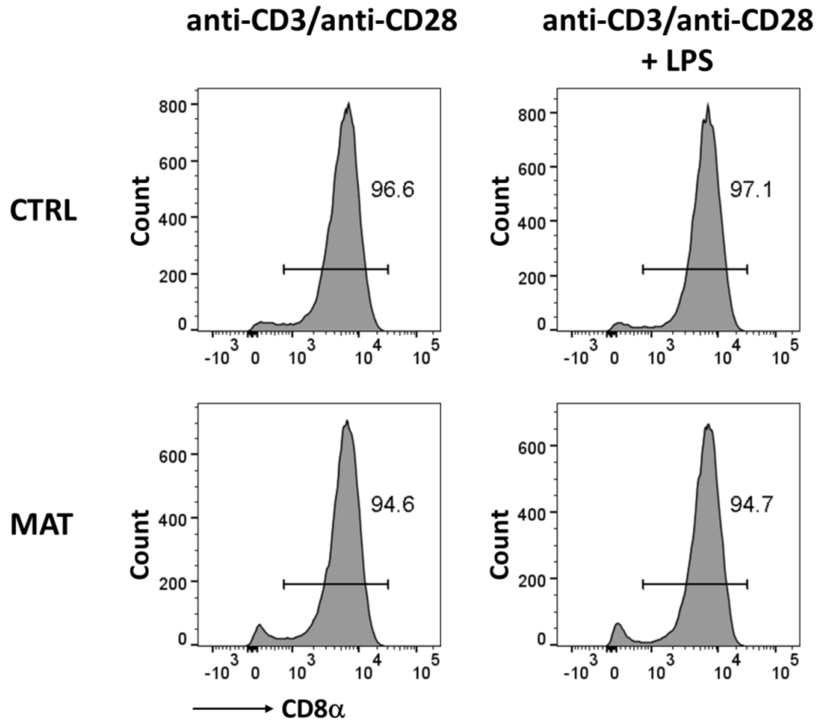

Supplementary Figure 3. CD8 T cell purity from MAT and CTRL effector T cells generated *in vitro* with or without LPS. Total $CD8^+$ T cells purified from gender-matched pooled spleens of uninfected dol 15 CTRL and MAT infant mice were stimulated with anti-CD3/anti-CD28 with or without *Escherichia coli*-derived LPS (1 μg/ml) for 72 hours. The percentage of $CD8^+$ T cells in these cultures is shown. Data is representative of three independent experiments.
